# Sclerosing Stromal Tumor of Ovary: A Case Report

**DOI:** 10.1155/2012/592836

**Published:** 2012-10-12

**Authors:** Menka Khanna, Ashish Khanna, Mridu Manjari

**Affiliations:** ^1^Department of Pathology, Sri Guru Ram Das Institute of Medical Sciences and Research (SGRDIMSR), Amritsar 143001, India; ^2^Department of Microbiology, Sri Guru Ram Das Institute of Medical Sciences and Research (SGRDIMSR), Amritsar 143001, India

## Abstract

Sclerosing stromal tumor (SST) is an extremely rare and distinctive sex cord stromal tumor which occurs predominantly in the second and third decades of life. We report a case of a 32-year-old woman who developed a sclerosing stromal tumor of ovary and presented with irregular menstruation and pelvic pain. Her hormonal status was normal but CA-125 was raised. She was suspected to have a malignant tumor on computed tomography and underwent bilateral salpingo-oopherectomy. It is therefore necessary to keep in mind the possibility of sclerosing stromal tumor in a young woman.

## 1. Introduction

Sclerosing stromal tumor (SST) is an extremely rare benign subtype of ovarian stromal neoplasm of the sex cord stromal category that has distinctive clinical and pathological features which differentiate it from other stromal tumors. The tumor occurs predominantly in the 2nd and 3rd decades of life [1, 2]. It is usually unilateral and well circumscribed; its recurrence has not been reported [3]. Histologically it is characterized by a network of thin walled vessels, sclerosis, heterogeneity of the cellular areas and ill defined cellular pseudolobules separated by a densly hyalinised or markedly edematous stroma [1].

## 2. Case Report

**Figure 1:**
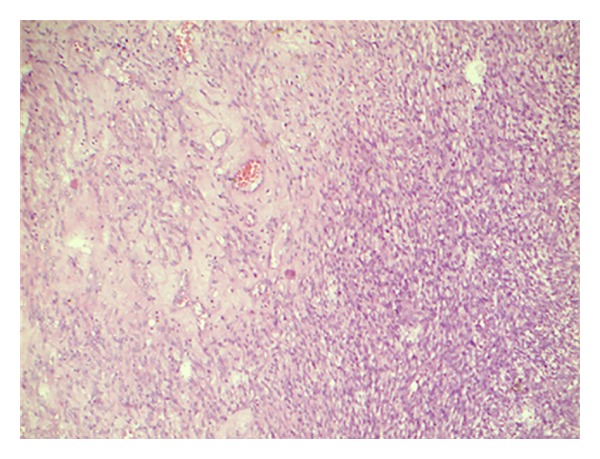
Hypercellularand hypocellular areas with prominent vascularity (H&E ×100).

**Figure 2:**
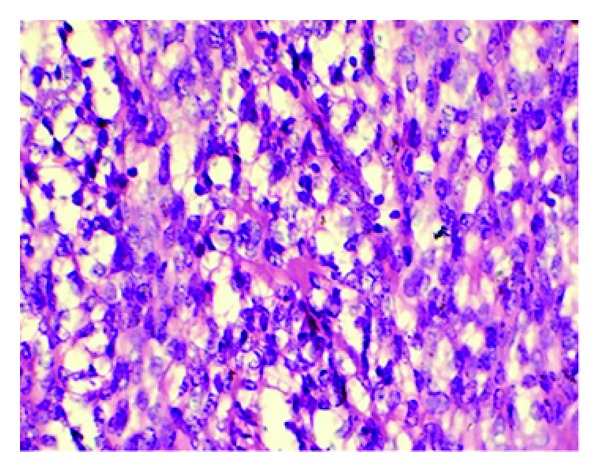
Tumor cells are of two populations. One population is of spindle cells. The other population of cells is round with clear cytoplasm (H&E ×400).

**Figure 3:**
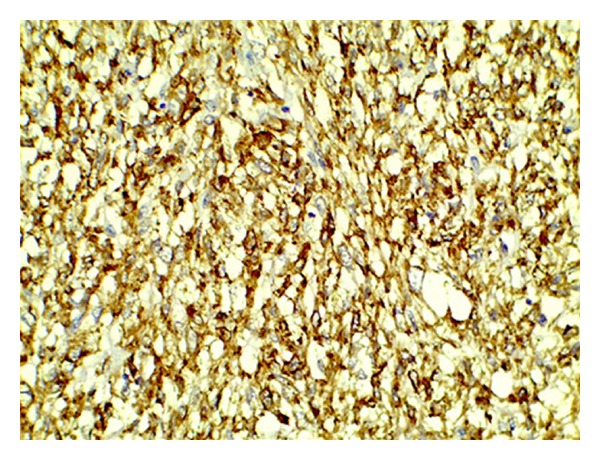
Tumor cells revealing diffuse immunoreactivity to smooth muscle actin (IHC for smooth muscle actin 400x).

A 32-year-old woman presented in the outpatient department of obstetrics and gynecology with menstrual irregularities and pelvic pain for 3 months and abdominal distension for one month. On clinical examination a large abdomino-pelvic mass was palpable. Ultrasonography showed a large heterogeneous, predominantly solid pelvic mass with some cystic foci and measured approximately 15 cm. Computerised tomography showed a large well-defined inhomogeneous solid and cystic lesion not separately defined from right ovary. No calcifications were seen. After IV contrast administration, the lesion showed inhomogenous enhancement (solid areas show moderate enhancement). The fat planes with urinary bladder were partially effaced. Radiological opinion was suggestive of malignant ovarian tumor. CA-125 level was 843 IU/mL. The patient was hospitalized with the diagnosis of a malignant ovarian tumor. All hematological investigations and serum hormonal assays were normal. Ascitis was not present. Bilateral salpingo-oopherectomy was done with no intraoperative pathologic diagnosis. The gross examination of the resected specimen showed an encapsulated 16 × 13 × 11 cm oval mass with attached ovary and stretched fallopian tube on its surface. The outer surface was smooth and intact. Cut section was grayish white solid with rubbery consistency and small cystic spaces. No hemorrhage or necrosis was observed. Microscopic examination showed a tumor with lobular pattern of growth having prominent interlobular fibrosis. Alternate hyper and hypocellular areas were seen with prominent vascularity (Figure 1). The lobules were composed of spindle-shaped cells along with round clear and signet ring like cells (Figure 2). Immunohistochemistry showed the cells to be positive for smooth muscle actin (Figure 3). A diagnosis of sclerosing stromal tumor of ovary was made.

## 3. Discussion

Sclerosing stromal tumor is a benign subtype of ovarian sex cord stromal tumor, described as a distinct entity in 1973 by Chalvardjian and Scully [1]. Sex cord stromal tumors represent approximately 8% of ovarian neoplasms [4] and SST comprises less than 5% of sex cord stromal tumors. This relatively rare tumor characteristically differentiates itself histologically and clinically from others. The common presenting symptoms of SST include menstrual irregularity, pelvic pain and non-specific symptoms related to ovarian mass. Masculinisation or anovulation may be present in some patients as they are occasionally associated with oestrogen and rarely androgen secretion. The patient reported here had no clinical virilisation; hormonal levels were normal but CA-125 was elevated. SST usually presents in the 2nd-3rd decade of life, whereas other ovarian stromal tumors present in the 5th-6th decade of life [5].

Microscopic picture of SST is heterogenous and contrasts with the relative homogeneity of other stromal tumors like thecoma and fibroma. Histologically it is characterized by cellular psuedolobules, prominent interlobular fibrosis, marked vascularity and dual cell population, collagen producing spindle cells, and lipid containing round or ovoid cells [6].

The vascular sclerotic and edematous stromal changes are constant features of these tumours and relate to the local elaboration of some vascular permeability and growth factors (VPF/VEGF) [7]. Vascular tumours are also included in the differential diagnosis due to prominent vascularity, but inhibin positivity suggests the diagnosis of sclerosing stromal tumour. Sometimes massive ovarian edema may be confused with sclerosing stromal tumours but can be differentiated by preserved ovarian tissue within edematous stroma and absence of heterogeneity. Moreover, the edema in sclerosing stromal tumor is zonal in contrast to that seen in massive edema or an edematous fibroma. Sometimes the edematous stroma of these tumors contains vacuolated cells and signet ring cells (as seen in our case) which can be mistaken for signet ring cells of Krukenberg tumour of ovary. But they can be differentiated as the latter are mostly bilateral, occur usually in the 6th and 7th decades and lack pseudo-lobular pattern of sclerosing stromal tumour. Furthermore, signet ring cells of Krukenberg tumour contain mucin rather than lipid and the cells may exhibit nuclear atypia and mitotic activity. Immunohistochemical analysis in SST shows positivity for predominant smooth muscle actin and inhibin and vimentin suggesting stromal origin of sclerosing stromal tumours [8]. 

Calcitonin, inhibin, CD34, and *α*-glutathione S-transferase (*α*GST) positivity has been reported to be useful to differentiate sclerosing stromal tumors from thecomas, fibromas and other sex cord stromal tumors. Inhibin has been shown to be useful marker for ovarian sex cord stromal tumors. CD34 stains the endothelium of often dilated and branching vascular architecture and clearly distinguishes SST from thecoma and fibroma. *α*GST positivity within scattered cells appears to be useful in the distinction of SST from diffuse staining thecomas and no staining fibromas [9].

On ultrasonography the appearances of SST may mimic that of malignant ovarian tumors because they show mixed pattern of cystic and solid components [10]. However color doppler ultrasonography of SST reveals prominent vascularity in the peripheral portion and central intercystic spaces [11]. Magnetic resonance imaging is more helpful in differentiating SST from malignant ovarian tumors which include a large mass with hyperintense cystic components or a heterogenous solid mass of intermediate-to-high signal intensity on T2 weighted MRI. Dynamic contrast-enhanced images can even distinguish SST from other sex cord stromal tumors with striking early peripheral enhancement reflecting cellular areas with prominent vascular networks and an area of prolonged enhancement in inner portion of the mass representing collagenous hypocellular area. These findings are not a feature of thecomas and fibromas. This shows that MRI is useful in making a preoperative diagnosis of SST and distinguishing SST from other malignant ovarian tumors as well as other stromal tumors [12].

Due to the rarity of this particular ovarian neoplasm it is not always possible to predict the presence of this tumor preoperatively on the basis of clinical and sonographic findings. But a possibility of sclerosing stromal tumor should be kept in young patients with ovarian mass, as all of the sclerosing stromal tumors of the ovary reported in the literature were benign and were treated successfully by enucleation or unilateral ovariotomy.
